# Characterisation of behaviours relevant to apathy syndrome in the aged male rat

**DOI:** 10.1016/j.bbr.2024.114977

**Published:** 2024-04-01

**Authors:** Megan G. Jackson, Stafford L. Lightman, Emma S.J. Robinson

**Affiliations:** a School of Physiology, Pharmacology and Neuroscience, University of Bristol, Bristol, UK; b Bristol Medical School: Translational Health Sciences, University of Bristol, Bristol, UK

**Keywords:** Apathy, ageing, animal model, behaviour, rat

## Abstract

Apathy is a complex psychiatric syndrome characterised by motivational deficit, emotional blunting and cognitive changes. It occurs alongside a broad range of neurological disorders, but also occurs in otherwise healthy ageing. Despite its clinical prevalence, apathy does not yet have a designated treatment strategy. Generation of a translational animal model of apathy syndrome would facilitate the development of novel treatments. Given the multidimensional nature of apathy, a model cannot be achieved with a single behavioural test. Using a battery of behavioural tests we investigated whether aged rats exhibit behavioural deficits across different domains relevant to apathy. Using the effort for reward and progressive ratio tasks we found that aged male rats (21–27 months) show intact reward motivation. Using the novelty supressed feeding test and position-based object exploration we found aged rats showed increased anxiety-like behaviour inconsistent with emotional blunting. The sucrose preference test and reward learning assay showed intact reward sensitivity and reward-related cognition in aged rats. However, using a bowl-digging version of the probabilistic reversal learning task, we found a deficit in cognitive flexibility in aged rats that did not translate across to a touchscreen version of the task. While these data reveal important changes in cognitive flexibility and anxiety associated with ageing, aged rats do not show deficits across other behavioural domains relevant to apathy. This suggests that aged rats are not a suitable model for age-related apathy syndrome. These findings contrast with previous work in mice, revealing important species differences in behaviours relevant to apathy syndrome in ageing.

## 1 Introduction

Apathy is a complex neuropsychiatric syndrome with symptoms spanning emotional, cognitive and behavioural domains [28,39]. The characterisation of these domains has shifted over the past two decades. More recently, the diagnostic criteria and list of apathy dimensions were revised, incorporating work stemming from a newly developed Apathy Motivation Index [3,39]. The behaviour-cognition domain incorporates symptoms relating to deficits in motivation to initiate/complete a task. The emotional domain relates to inability to appropriately react to events that have either positive or negative valence. The social domain relates to deficits in initiating/maintaining social contact. To be diagnosed with clinical apathy, the individual must show behaviours relating to at least two apathy dimensions i.e., behavioural/cognitive, emotional, or social motivation for a period of at least four weeks.

Apathy accompanies a broad range of neurodegenerative, neurocognitive and psychiatric disorders, but also emerges in otherwise healthy ageing. A study showed that in a population of 76 healthy, community-dwelling elderly subjects, apathy increased with age over a 5-year period [8]. Cross-sectional analysis of an elderly, dementia-free population of 4354 participants showed that 49% of them had ≥ 2 symptoms of apathy. Those with apathy symptoms were significantly older than those who did not [19]. Apathy is associated with higher mortality, poorer quality of life, cognitive decline and significant caregiver stress [17,23]. It can also negate the positive effects of therapeutic intervention by affecting adherence to medication and healthcare appointments [35]. Despite its clinical relevance and research importance, the underlying neurobiology of apathy remains unclear, and does not yet have a designated treatment strategy.

Generating a translational animal model for apathy could allow for potential treatments to be tested and mechanisms to be elucidated. However, comprehensive models of apathy syndrome are so far limited and most studies rely on a single behavioural test. Given the multidimensional nature of the syndrome, and the diagnostic criteria relating to development of symptoms across two domains of apathy, an apathy model is unlikely to be achieved with a single behavioural test. At the preclinical level, a deficit in motivation for reward alongside evidence of emotional blunting represent behavioural hallmarks of apathy behaviour and distinguish it from behaviours relating to major depressive disorder, which is often characterised by negative affective state and exaggerated response to negative stimuli [5,25]. In a behavioural battery, previous work has shown that aged mice show deficits in physical effort-based motivation for reward in the effort for reward (EfR) and progressive ratio (PR) tasks and evidence of emotional blunting (reduction in anxiety response, reduced corticosterone response and brain activation in stress areas following a restraint stressor) [24] suggesting natural ageing could be used as a model of apathy. In addition to physical effort and emotional processing changes, patients may experience cognitive symptoms, including a reduction in cognitive flexibility, planning and decision making [29]. The probabilistic reversal learning task (PRLT) provides a translational measure of cognitive flexibility and feedback sensitivity used across species [4,13]. In this paradigm, subjects must adjust their behaviour to changing contingencies. This task is sensitive to deficits in cognitive flexibility in conditions where apathy commonly occurs, such as Parkinson’s Disease [36] and as such, use of this task may be a relevant measure of cognitive deficits associated with apathy syndrome at the preclinical level. The use of a multidimensional approach allows for the phenotype to be more meaningfully integrated across domains and dissociated from behavioural profiles observed in other disease states.

While we have previously demonstrated that aged mice may be suitable to assess certain behaviours relating to distinct apathy domains, relevant cognitive changes have not been well characterised. Rats may be a more suitable species for tasks of this nature given their greater propensity to reverse in standard probabilistic paradigms and more established repertoire of cognitive tasks [32]. Therefore, in this study we combined tests of reward motivation (including the effort for reward, progressive ratio task) and emotional processing (novelty supressed feeding test (NSFT), restraint stress, position-based object exploration, reward learning assay (RLA), sucrose preference test (SPT)) in addition to measures of cognitive flexibility (probabilistic reversal learning) to investigate whether aged rats exhibit behavioural deficits across different domains relevant to apathy syndrome. For the purpose of this study we defined aged rats as animals more than 21 months old.

Given that apathy syndrome is characterised by a reduction in motivated behaviour, emotional blunting and impaired cognitive flexibility [25], we hypothesised that we would observe a similar pattern of deficits in aged rats. Relating to the emotional domain, we predicted a reduced response in hyponeophagia in the NSFT, reduced stress-induced corticosterone and reduced bias in the position based object exploration test indicative of negative affective sensitivity. In addition, we expected to see a reduction is sucrose preference (SPT) and reward bias (RLA) indicative of reduced positive affective processing. Relating to the behaviour-cognition domain we expected a reduction in motivation in the effort for reward and progressive ratio tasks, and an impairment in the PRL tasks. Deficits in at least two of these apathy domains would be consistent with clinical diagnosis [39] and indicative of an apathy phenotype rather than isolated age-related impairments in behaviour.

## 2 Methods

### 2.1 Subjects

10 aged, male Sprague-Dawley rats (aged 21 months old at start of experiments, weighing 467–530 g and 25 months, weighing 480–555 g by end) and 10 sex and strain-matched younger controls (4 months old and weighing 310–320 g at start of experiments, 8 months, weighing 470–545 g by end) were kindly supplied by Eli Lilly. Sample size selected was suitable for detecting larger effect sizes detected in a similar ageing study [24] using G*power 3.1.9.7 (α error probability = 0.05, power (1-β) 0.85). Sample size was calculated using effect sizes from the following tasks also used in the present study: the effort for reward task (Cohen’s *d* = 2.078, suggested sample size n = 5/group), the novelty supressed feeding task (*d* = 1.391, suggested sample size n = 9/group), sucrose preference test (*d* = 1.343, suggested sample size, n = 9/group), progressive ratio task (*d* = 1.863, n = 5/group). We took the largest number from these calculations (n = 9) and due to group housing conditions for optimal rat welfare we chose n = 10. It should be noted that this number may be too low to detect more subtle changes in behaviour, or tasks where we did not have appropriate data to calculate effect sizes.

The aged rats had spent a period of approx. 5 months on a restricted diet (16 g fed between ZT2–3) at Eli Lilly to prevent excessive weight gain and promote good welfare/healthy ageing [16]. This was continued for a period of 5 days at Bristol to aid adjustment before being put on *ad libitum* feeding for the beginning of experiments. Rats were housed in enriched cages, consisting of a red shelf, wooden chew block and cardboard tube in groups of either threes or pairs (counterbalanced across age groups). Water and food were given *ad libitum*, unless undergoing operant training, novelty supressed feeding testing or sucrose preference. In the case of food-motivated behavioural testing, a regime of 18 g/rat was used. They were kept in 12:12 reverse lighting, with lights OFF at 8.15 am and ON at 8.15 pm. Behavioural testing was conducted under red lighting and within the animal’s active phase. Due to age being the factor of analysis, rodents could not be fully randomised to groups. However, within a behavioural study, order of testing was counterbalanced across age to mitigate time of day effects. Aged animals were checked daily for general health and any impairments which could confound interpretation of behavioural studies. We implemented a health-related exclusion criteria where any animal which showed signed of declining health was removed from the study. This included rapid/unexplained weight loss, and decline of motor function of the back legs. No animals were excluded on this basis. It should be noted that behavioural tests were performed sequentially and therefore there is the potential that advancing age differentially affected some measures (timeline provided in supplementary figures, S1). A summary of the behavioural tasks and associated behavioural domains tested are provided in Table 1. To help reduce confounds associated with the order of the behavioural tasks, the design randomised the tasks between measures of motivation-related and emotional behaviour. We prioritised spontaneous tasks before operant training tasks to mitigate potential impacts of prolonged conditioning training on natural, spontaneous behaviour. We ran restraint stress at the end of the behavioural battery to ensure acute stress experience did not impact on subsequent behavioural testing. Due to clear visual differences between young and aged rats, the experiment could not be blind to age while running behavioural experiments. All experiments took place in the animals’ active phase and were performed in accordance with the Animals (Scientific Procedures) Act (United Kingdom) 1986 and were approved by the University of Bristol Animal Welfare and Ethical Review Body (AWERB).

To further manage the impacts of health impairment confounding behaviour interpretation, a second cohort of rats were brought in (supplementary figures, S1). Here, a further 10 aged male Sprague-Dawley rats were obtained from Eli Lilly (aged 23 months at experiment onset, weighing 440–525 g and aged 27 months and weighing 401–560 g by experiment end). Rats arrived at the Bristol unit aged 21 months but due to COVID restrictions experiments did not start until 2 months later. During this delay period aged rats were fed a restricted diet of 18 g/rat. One week before experimentation started aged rats were put onto *ad libitum* feeding to match young controls. 10 strain and sex-matched controls were obtained from Envigo at a weight between 300 and 324 g, aged approx. 3 months and aged 7 months and weighing between 403 and 460 g by experiment end. As above, rats were provided with *ad libitum* food and water unless undergoing food or water motivated behavioural testing. In the case of food-motivated tasks, a restricted diet of 18 g/rat was resumed.

### 2.2 Object exploration

Rats were placed in a square arena (1 m x 1 m) lined with sawdust. They were allowed to explore the arena freely under red lighting for a period of 15 min. The following day, a novel object (either a pinecone or a scrubbing brush) was placed in either the centre (higher risk) or to the side of the arena (lower risk). The rat was placed in the arena and could explore the arena and novel object for a period of 10 mins. This test is based on the well-characterized principle that a rodent will exhibit thigmotaxis when placed in an open arena [20]. As such, the rodent should bias its exploration of an object positioned to the side of the arena versus the object placed in the center. Sessions took place over two days, and position and object were counterbalanced over these two sessions and across age group. Their movement was recorded using a Logitech webcam, and the videos were subsequently coded so that the experimenter was blind to age group. Time spent exploring the object and bouts of exploration were manually recorded, and a % object placement preference was calculated.

### 2.3 Novelty supressed feeding test

Rats were food restricted for 24 hrs before onset of the test. During testing, rats were placed in a circular arena (70 cm in diameter), lined with sawdust. The test was conducted under white lighting, but during the animals’ active phase. A bowl was placed in the centre of arena containing standard lab chow. Time taken for the rat to approach the bowl and to eat from it was manually recorded using a stopwatch. Sawdust was redistributed between rats and the bowl was washed and fresh food pellets were used each time. Time taken for the rats to eat from a centrally-placed bowl is used as a measure of anxiety-like behaviour. The longer the rat takes to overcome its aversion to an open space to eat from the bowl, the greater its level of anxiety-like behaviour. The additional measure of time taken to approach the bowl is used to test whether there are differences in detecting the bowl which may be driven by other potential other factors that may relate to general impairment.

To test for potential age-related appetite confounds that may drive results observed in the novelty supressed feeding test, rats were food restricted for 24hrs. They were then placed in individual test cages they had previously been habituated to, with a known weight of food. After a period of 10 mins, total amount eaten in g was measured. Rats were then returned to their standard feeding regime.

### 2.4 Lever training and fixed ratio testing

Rats were trained in sound-proofed operant boxes (Med Associates Inc) which were run on Klimbic software (Conclusive Solutions Ltd., UK) similar to the protocol previously described by [18]. Each box consisted of two levers positioned either side of a reward magazine which was connected to a reward pellet dispenser (45 mg rodent tablet, TestDiet, Sandown). Only one lever was active during training/testing, and positioning was counterbalanced across cohorts. Unless otherwise stated, a session lasted 30 mins. Rats first learned to associate the magazine with the delivery of reward where pellets were dispensed automatically every 40 s for two sessions. Rats then underwent three sessions of continuous reinforcement learning, in which they learned to associate a lever press with the delivery of a reward. They then progressed on to fixed ratio (FR) training. FR refers to the number of lever presses to receive one reward pellet. Rats progressed through FR1, 2, 4 and 8 after two consecutive sessions of 100+ trials, and then FR16 after two consecutive sessions of 70+ trials. All rats then underwent at least 5 additional FR16 sessions.

### 2.5 Effort for reward task

Directly following lever training and testing up to FR16, rats underwent a single session of the EfR task, previously described by [18,41]. Here, rats underwent a standard FR16 session, with the addition of a bowl of powdered standard lab chow placed in front of the inactive lever. The chow was accessed through a small hole in the lid. The FR16 requirement for a reward pellet represents the high effort, high value reward option, while the freely available lab chow represents the low effort, low value reward option. Throughout the 30 min session the animal could freely choose to work for reward or consume the chow. At a later timepoint in the experimental timeline, rats completed 5 consecutive EfR sessions under both food restriction and *ad libitum*. The primary output measures for this task were the number of high effort, high reward trials, later termed ‘trials’, and consumption of the low effort, low value reward (standard powdered lab chow).

### 2.6 Progressive ratio task

Rats were next tested in the PR task. In this task, the rat must make an increasing number of lever presses for each successive reward. We used a response schedule of F(*n*) = 5 × EXP(0.2 *n*)−5 [24,40]. The session lasted a maximum of 60 minutes. The response ratio completed before the rat stopped responding within the session time limit for a period of 5 minutes was used as a breakpoint, and this metric is used as readout of motivational state. In some cases, rats continued until the max time limit and this was termed ‘final ratio completed’ and was analysed separately from breakpoint. A separate PR test session was conducted under food restriction conditions and *ad libitum* feeding. The primary output measures for this task were breakpoint and/or final ratio completed.

### 2.7 Restraint stress and corticosterone analysis

Rats were restrained for 30 mins using a restraint tube (52–0292 RODENT RESTRAINER, Biochrom). Blood samples were collected immediately preceding and post-stress. Blood was collected by inserting a butterfly needle into the tail vein. Blood was dripped into an Eppendorf containing 50 µL EDTA. Blood samples were then centrifuged at 8000 rpm for 10 mins. Plasma was collected and stored at −20 °c until analysis. A radioimmunoassay (RIA) was performed in-house to measure total corticosterone from restraint stress blood samples as previously described [55]. Samples were analysed by RIA in triplicate to account for variation in assay results and the median value of the triplicate was used in analysis. See supplementary methods for more details. The primary output measure was corticosterone (ng/ml) pre- and post-stress.

### 2.8 Sucrose preference test

During habituation, rats were water restricted for a period of 4 h and were then placed in test cages containing two sipper sacks (Avidity Science) for 1 h. On the first and third day the sipper sacks contained 2% sucrose, and on the second day they contained tap water. On the test day, the rat was presented with one sipper sack containing 2% sucrose and the other containing tap water. Starting position of sucrose was counter balanced and swapped at 30 mins. Amount consumed (ml) was measured at each time point and a % sucrose preference was calculated as primary output measure using: (amount of sucrose solution consumed/total solution consumed) x 100.

### 2.9 Bowl digging training

Rats were first habituated to a 40 cm×40 cm Perspex arena over two days and underwent bowl digging training where they learnt to retrieve a food reward buried under increasing amounts of sawdust as outlined in [47] and described in further detail in supplementary methods. Following successful digging training rats underwent a single discrimination training session, where the rat was presented with two bowls filled with 2 cm of a different digging substrate e.g., sponge, perlite, cardboard squares etc. One of these substrates was consistently baited with one reward pellet (CS+) and the other was not rewarded (CS-). Both substrates were mixed with a crushed reward pellet to prevent odour discrimination. In the first trial, the rat explored both bowls until the pellet was found. In the second trial, as soon as the rat chose one bowl, the other was removed. If the rat chose the baited bowl, it was recorded as correct. The criterion for a successful choice was the rat digging in the correct bowl (nose in contact with substrate and paws moving the substrate). This was repeated until the rat chose the baited bowl over 6 consecutive trials within a maximum of 20 trials. After this, the rat was considered fully trained and ready for testing. n = 1 aged rat did not meet criteria for bowl digging training and was not tested in the bowl digging paradigms.

### 2.10 Reward learning assay

**Fig. 1:**
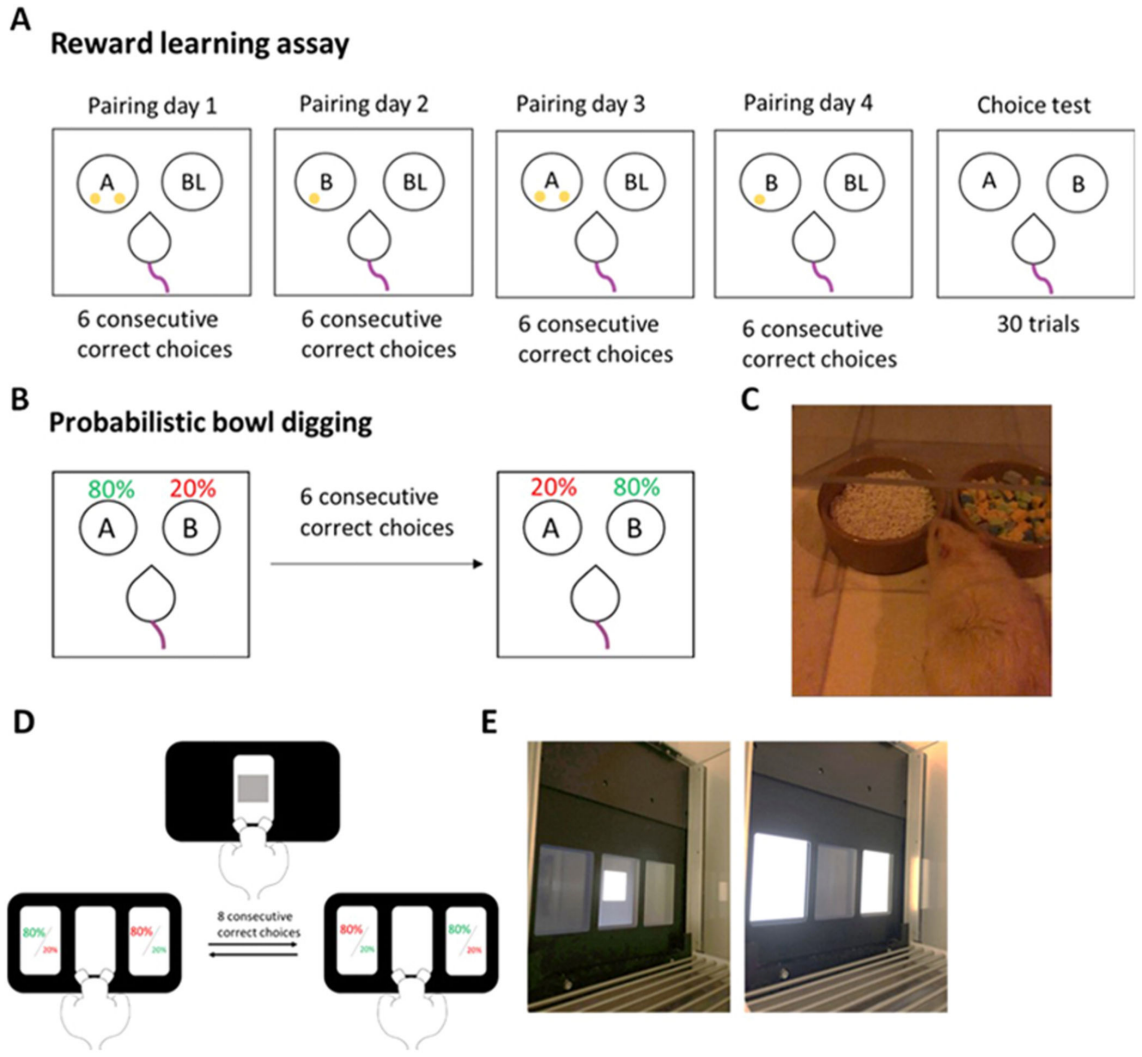
Reward learning and probabilistic reversal learning tasks. **A** The reward learning assay which consists of 4 pairing sessions followed by a choice test. **B** The probabilistic bowl digging task. **C** An aged rat making a digging decision. **D** The rat presses an initiation square and then must select either the left or right window, which are rewarded either 80% or 20% of the time. **E** View from operant box. A = substrate A, B = substrate B, BL = blank substrate.

The RLA is a 5 consecutive-day protocol consisting of 4 pairing sessions followed by a testing day as previously described by [47]. Each pairing session followed the same protocol as the discrimination session above (CS+ vs CS-). The rat learned two independent reward associations (CSA+ or CSB+), where one substrate was associated with one reward pellet (+) and the other with two pellets (++). Over the course of the 4 pairing sessions, the rat is presented twice with substrate reward contingencies (CSA+ vs CS- or CSB++ vs CS-). On the testing day, the rat was presented with the two previously reinforced substrates (CSA vs CSB) (Fig. 1). The substrates were randomly reinforced with one reward pellet (probability 1 in 3 CSA or CSB) over the course of 30 trials. Each substrate was mixed with a crushed pellet to prevent odour discrimination. The position of the substrates, left or right, was pseudorandomised. Substrate choice over the 30 trials and number of pellets consumed was recorded. % choice bias for the two-pellet paired substrate was the primary output measure and was calculated using (number of two-pellet paired choices/30*100)-50. A positive value indicates a reward -induced positive bias. Additional checks for substrate bias, directional bias and number of pellets consumed relative to chance were performed.

### 2.11 Probabilistic reversal learning bowl digging task

In a single session, the rat was presented with two bowls containing two different substrates. One substrate was baited with a single pellet 80% of the time (‘rich’ substrate), and the other substrate baited 20% of the time (‘lean’ substrate). The rat had to choose the correct (rich) bowl 6 times consecutively within a maximum of 30 trials to successfully ‘acquire’ the first rule. Once the rat had acquired this initial rule the contingencies were reversed such that the rich substrate became the lean substrate and *vice versa*. The rule was reversed every time the rat chose the rich bowl 6 times consecutively within 30 trials (Fig. 1). The position of the bowls was swapped pseudo randomly as described in the RLA. The session ended after a maximum of 1 hr, or 4 consecutive omissions, or the rat failed to learn a rule within 30 trials. Successful learning of the first rule was termed ‘acquisition’ and successful learning of the new rule was termed a ‘reversal’. The primary output measures were number of reversals, win-stay probability and lose-stay probability, **summarised in** Table 2.

### 2.12 Probabilistic reversal learning operant task training

The operant box used for this task consisted of an infrared touchscreen panel with three windows where the animal could respond by touching them. A magazine was positioned centrally opposite the touchscreen panel where rats could access 45 mg reward pellets (Test-Diet, Sandown Scientific, UK). The box also contained a house light and tone generator. The task was set up as previously described in [54], which was based in the original design used in [4]. Rats first underwent a 30 min session where a reward pellet was dispensed into the magazine automatically every 40 s. Rats then underwent continuous reinforcement training where touching the initiation square in the middle window resulted in the delivery of a reward pellet. This was accompanied by a tone and the magazine illuminating. The session finished after a maximum of 30 mins or the rat completed 120 trials. Once rats completed 120 trials a day for two consecutive days, they progressed to a second continuous reinforcement stage, where the rat was required to respond to the initiation square and then the illuminated window either to the left or right to receive a reward pellet. The session finished after a maximum of 40 mins or the rat completed 200 trials. If the rat touched the initiation square but took >10 s to respond to the left or right panel, then a 10 s timeout occurred where the house light came on and no reward was delivered. Once the rat had completed >120 trials over two consecutive sessions it was deemed trained and ready to undergo the probabilistic reversal learning task.

### 2.13 Probabilistic reversal learning task test sessions

One window was designated the ‘rich’ stimulus and was rewarded 80% of the time (delivery of reward pellet) and punished 20% of the time (10 s timeout and house light on). The other window was designated the ‘lean’ stimulus and was punished 80% of the time and rewarded 20% of the time. The starting position of the rich stimulus was counterbalanced across cohort and remained consistent across sessions. When the rat made 8 consecutive rich choices, the contingencies swapped, such that the window that was previously designated rich became lean and vice versa (Fig. 1). The contingencies continued to change each time the new rich stimulus was selected 8 consecutive times up to a maximum of 40 mins or 200 trials.

### 2.14 Statistical analysis

In this study design, the single animal was considered the experimental unit. Data was tested for normality using the Shapiro-Wilk test. Where data were normally distributed, data were analysed with an independent t-test, or RM two-way ANOVA/mixed model ANOVA for non-statistical missing values where performance across sessions or phases was additionally analysed. Where normality was violated, the Mann-Whitney U test was utilized. Significance was defined as p < 0.05. Where significant main effects or interactions were found these were reported in the results and followed up with appropriate post-hoc tests. Where a p value of < 0.1 was found it was reported in the results as a trend level effect but not further analysed. *A priori* and consistent with other work [30], statistical outliers were defined as data points 2 standard deviations from the group mean. Where this occurred, these points were excluded, and replaced with the group mean to allow repeated measures analysis where required. If a rat had two outlying data points across sessions then the animal was excluded from the analysis for that measure. Instances where this occurred are reported in a table in supplementary figures (S2). Statistical analysis and graphing were conducted using GraphPad Prism v 10.0 for windows.

## 3 Results

### 3.1 Aged rats do not show impairment in physical effort-related choice behaviour

**Fig. 2:**
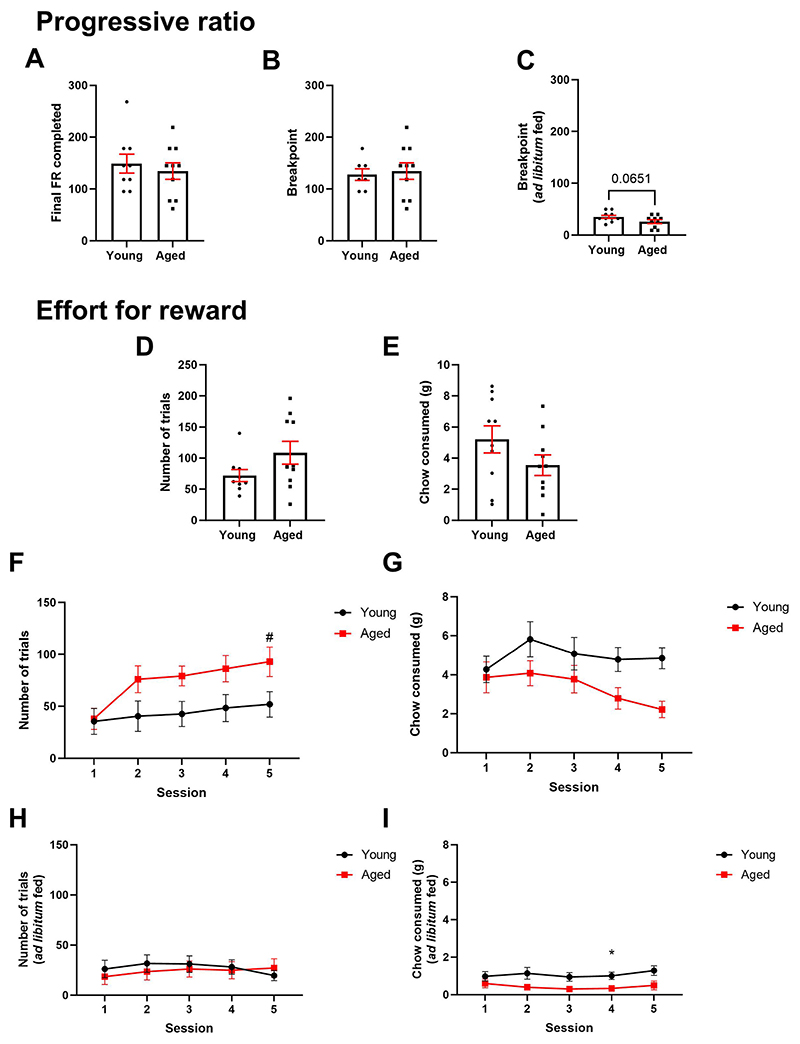
Aged rats do not show any motivational impairments in the progressive ratio or effort for reward tasks. **A** Under food restricted conditions, there was no difference in final ratio completed or **B** breakpoint in young versus aged rats (p > 0.05). **C** Under ad libitum feeding conditions there was a trend towards aged rats showing a reduced breakpoint. **D** In a single session of EfR directly after training, there was no difference in number of trials completed or **E** chow consumed (p > 0.05). **F** When repeated over 5 sessions, aged rats showed an increase in number of trials completed at day 5 vs day 1 (p < 0.05) **G** but no change in chow consumed (p > 0.05). Under ad libitum feeding conditions, there was no effect of age or session on number of trials (p > 0.05) **I** however aged rats consumed more chow on day 4 than younger rats (p < 0.05). *p< 0.05 (between-subject analysis) #p< 0.05 (within-subject analysis). Bars are mean ± SEM unless non-parametric where median ± interquartile range was used.

Rat performance in the progressive ratio task was analysed under food restricted and *ad libitum* feeding conditions. Under food restriction, not all rats reached a breakpoint and instead the last fixed ratio was used. There was no difference in last fixed ratio completed between age groups (p > 0.05) (Fig. 2.A). When only rats that reached a breakpoint were analysed, there was similarly no difference between groups (p > 0.05) (Fig. 2.B). Under *ad libitum* feeding conditions, there was a trend towards a lower breakpoint in aged rats (t_(18)_ = 1.964, p = 0.0651) (Fig. 2.C).

Rats were also tested in the effort for reward task, both directly after training in a single session, and over five consecutive sessions later in the behavioural battery. In the single session, there was no difference in number of trials completed or chow consumed (p > 0.05) (Fig. 2.D&E). When repeated over five sessions, there was a session*age interaction (F_(4,64)_ = 4.070, p = 0.0053). There was also a main effect of session (F_(2.126, 34.01)_ = 12.01, p < 0.0001) and a trend towards a main effect of age (F_(1,16)_ = 3.643, p = 0.0744). Post-hoc analysis revealed that number of trials completed by aged rats increased across sessions compared to session 1 (p = 0.0099–0.0162). Post hoc analysis of age comparison across sessions did not reach significance (p > 0.05) (Fig. 2. F). Analysis of chow consumed across sessions revealed a main effect of session (F_(2.587, 46.57)_ = 3.0, p = 0.0469). However, post hoc analysis did not reach significance. There was also a trend towards a main effect of age (F_(1,18)_ = 4.375, p = 0.0509) (Fig. 2.G). Under *ad libitum* feeding conditions, there was no effect of session or age on number of trials (p > 0.05) (Fig. 2.H). However, there was a main effect of age on chow consumed (F_(1,18)_ = 7.178, p = 0.0153). Post-hoc analysis revealed that younger rats ate more chow than aged rats on session 4 (Fig. 2.I).

### 3.2 Aged rats do not show behaviours or physiological changes consistent with emotional blunting

**Fig. 3:**
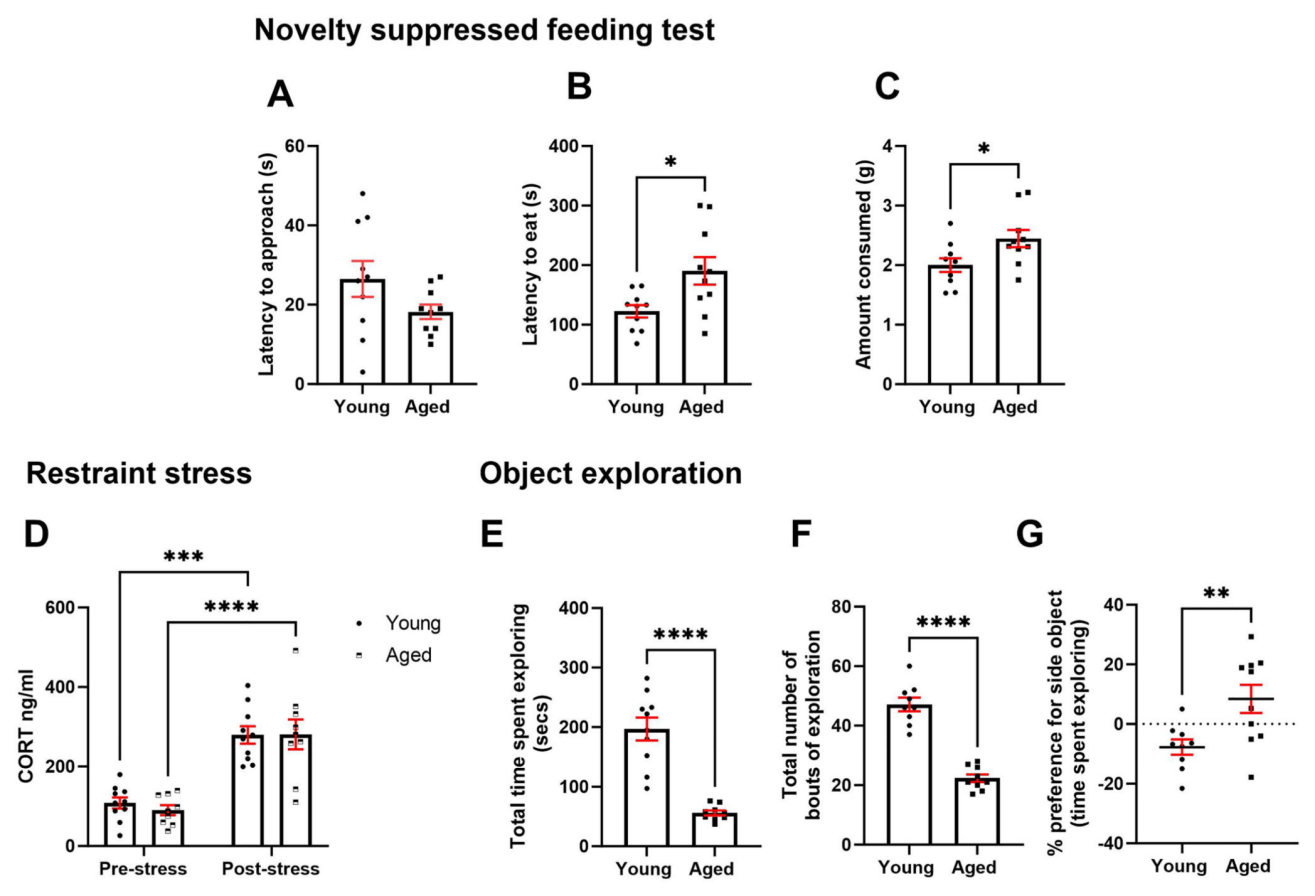
Aged rats do not show behaviours or physiological changes consistent with emotional blunting. **A** There was no age difference in latency to approach the centrally placed bowl in the NSFT (p > 0.05). **B** Aged rats took longer to eat from the bowl (p < 0.05). **C** Aged rats consumed more chow in a 10 min consumption test than young rats (p < 0.05). **D** Both young and aged rats showed an increase in blood plasma CORT levels following restraint stress. **E** Aged rats spent less time and **F** bouts of exploration of novel objects than young rats. **G** Aged rats showed a greater preference for the side placed object compared to younger rats (p < 0.05). *p< 0.05, **p<0.01, ***p< 0.001, ****p<0.0001. Bars are mean ± SEM unless non-parametric where median ± interquartile range was used.

Young and aged rats underwent a range of tests relating to negative affective sensitivity. In the novelty suppressed feeding test, there was no difference in latency to approach the bowl of chow (p > 0.05), but aged rats took longer to eat from the bowl than younger rats (t_(18)_ = 2.664, p = 0.0158) (Fig. 3.A&B). In a consumption test, aged rats consumed more lab chow within 10 minutes than younger rats (t_(18)_ = 2.416, p = 0.0266) (Fig. 3.C). Analysis of blood plasma corticosterone levels before and after restraint stress revealed a main effect of stress (F_(1,17)_ = 60.74, p < 0.0001) but no effect of age or stress*age interaction. Post-hoc analysis showed that plasma corticosterone was higher following stress in both young and aged rats (p = 0.0001 and p < 0.0001 respectively) (Fig. 3.D).

In a position-based exploration test, aged rats spent less time and had less bouts of exploring objects (t_(17)_ = 6.789, p < 0.0001 and t_(16)_ = 9.357, p < 0.0001) (Fig. 3.E&F). In addition, aged rats showed a greater preference for exploring an object placed at the side versus in the middle of an open field arena (t_(17)_ = 2.192, p = 0.0097) (Fig. 3.G). Aged rats showed no general impairment in locomotion in an open field test, p > 0.05 (supplementary figures, S3).

### 3.3 Aged rats show intact reward-related cognition and reward sensitivity but impaired cognitive flexibility in a bowl-digging version of the probabilistic reversal learning task

**Fig. 4:**
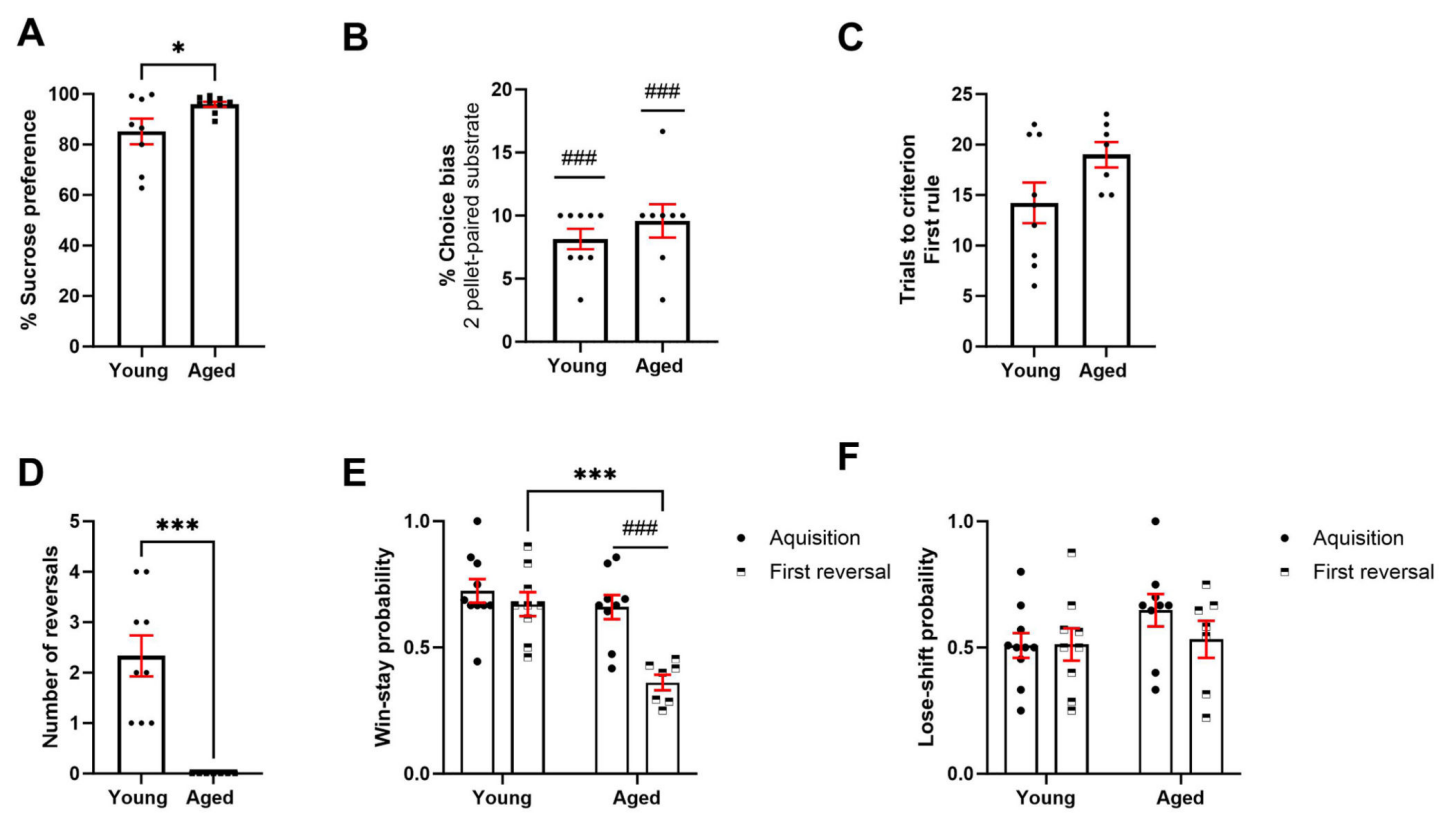
Aged rats show intact reward-related cognition and reward sensitivity but impaired cognitive flexibility in a bowl-digging version of the PRLT. **A** Both groups show a preference for sucrose, but aged rats show a greater preference than younger rats (p < 0.05). **B** Both groups showed a preference for the 2 pellet paired substrate in the RLA (p < 0.001) but no difference between age groups. **C** There was no difference in number of trials taken to learn first rule in the PRLT (p > 0.05). **D** Aged rats reversed less than younger rats in the PRLT (p < 0.001). **E** In the acquisition phase, aged rats had a lower win-stay probability than young rats (p < 0.001) and also had a lower win-stay probability in the reversal versus acquisition phase (p < 0.001). **F** There was no effect of age or phase on lose-shift behaviour (p > 0.05). *p< 0.05 (between-subject analysis) #p< 0.05 (within-subject analysis). Bars are mean ± SEM unless non-parametric where median ± interquartile range was used.

In the sucrose preference test, while both young and aged rats showed a preference for 2% sucrose solution over standard tap water (p < 0.001), aged rats showed a greater % sucrose preference than younger rats (t_(15)_ = 2.191, p = 0.0447) (Fig. 4.A). Aged rats also consumed more liquid overall than younger rats (t_(16)_ = 3.665, p = 0.0021) (supplementary figures, S4). In the reward learning assay, both young and aged rats showed a preference for the two-pellet paired substrate (t_(8)_ = 10.09, p < 0.0001 and t_(7)_ = 7.22, p = 0.0002 respectively) but there was no difference between groups (p > 0.05) (Fig. 4.B). In the probabilistic reversal learning bowl digging assay, there was a trend towards aged rats taking more trials to learn the first rule (t_(14)_ = 1.876, p = 0.0817) (Fig. 4.C). Of the rats tested, only rats that successfully acquired the rule were included in this analysis (n = 1 young rat and n = 2 aged rats did not acquire the first rule). When considering the rats that did acquire the first rule, aged rats completed less complete reversals than young rats (t_(14)_ = 5.001, p = 0.0002) (Fig. 4.D). Aged rats did not successfully reverse however still completed up to a max of 30 trials in the reversal phase. These trials were used to calculate win-stay/lose-shift probabilities in the reversal phase. Analysis of win-stay probability showed a main effect of age (F_(1,17)_ = 12.21, p = 0.0028), a main effect of phase (F_(1,14)_ = 24.42, p = 0.0002) and an age*phase interaction (F_(1,14)_ = 10.56, p = 0.0058). Post-hoc analysis revealed that young rats showed a higher win-stay probability in the reversal phase compared to aged rats (p = 0.0002). In addition, aged rats showed a reduction in win-stay probability in the reversal phase versus the acquisition phase (p = 0.0002) (Fig. 4.E). There was no effect of age or phase on lose-shift behaviour (p > 0.05) (Fig. 4.F).

### 3.4 Aged rats show no impairment in an operant touchscreen version of the probabilistic reversal learning task

**Fig. 5:**
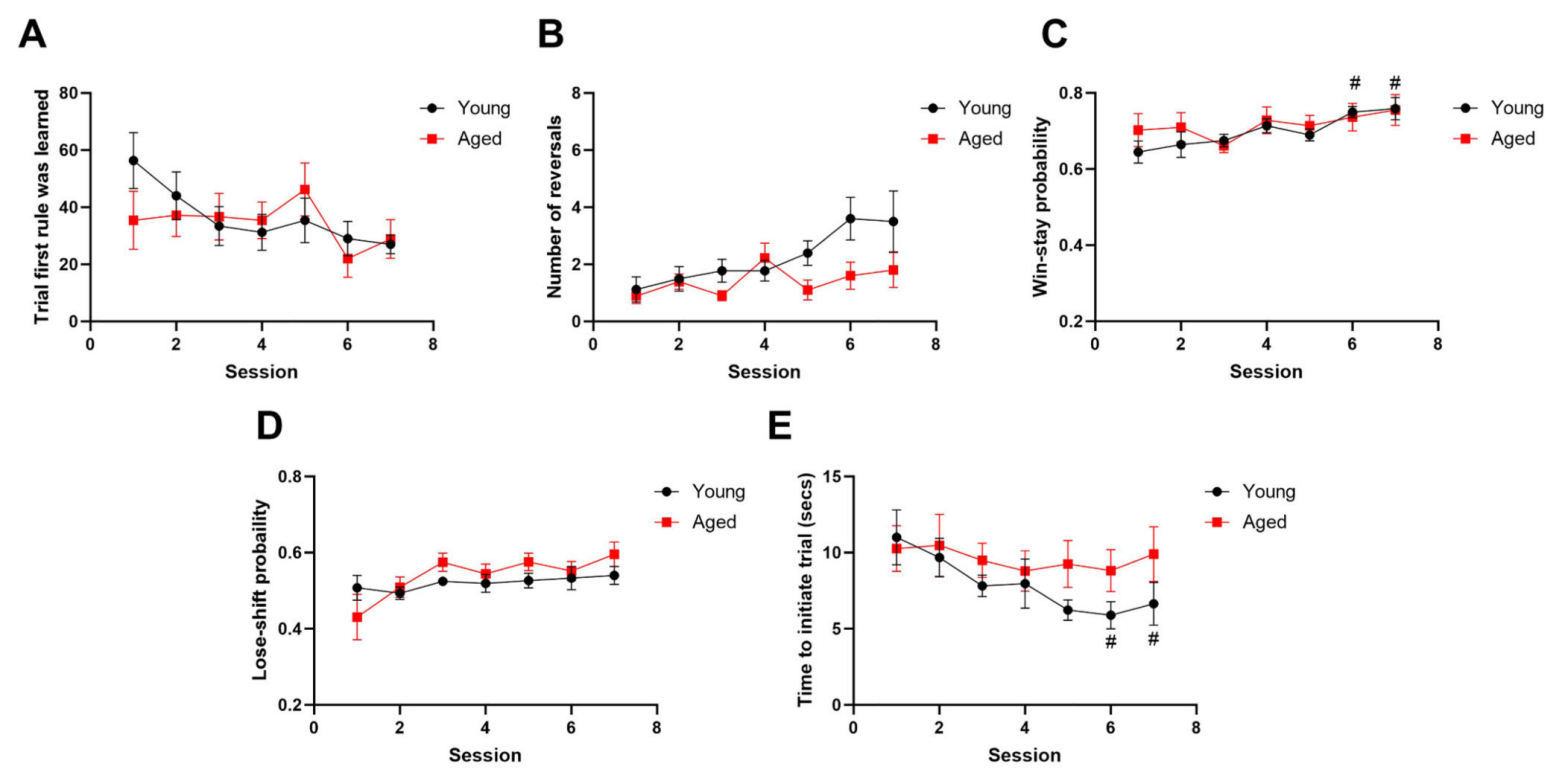
There was no effect of age on cognitive flexibility in the operant version of the PRLT. **A** There was no post-hoc effect of age on trial first rule was learned or **B** number of reversals (p > 0.05). **C** There was no effect of age on win-stay probability however win-stay probability was increased in the young group at session 6 and 7 versus 1 (p < 0.05). **D** There was no post-hoc level effect of age or session on lose-shift probability. **E** There was no effect of age on trial initiation time, however young rats showed a lower initiation time at session 6 and 7 versus 1. #p<0.05 (within-subject comparison). Points are mean ± SEM.

Of the rats that successfully learned the first rule, there was no effect of age or session on the trial at which the first trial was learned (p > 0.05) (Fig. 5.A). However, there was a main effect of session (F_(3.251, 55.81)_ = 5.719, p = 0.0013) and a session*age interaction (F_(6, 103)_ = 2.803, p = 0.0137) on number of reversals. However post-hoc analysis revealed no differences across session or age (p > 0.05) (Fig. 5.B). There was also a main effect of session on win-stay probability (F_(3.454, 62.17)_ = 4.219, p = 0.0064) but no effect of age (p > 0.05). Post-hoc analysis revealed that win-stay probability was increased at session 6 and 7 compared to session 1 in the young group (p = 0.013 and p = 0.040 respectively) (Fig. 5.C). There was a main effect of session on lose-shift probability (F_(3.07, 52.19)_ = 3.152, p = 0.0315) but no effect of age. However, post-hoc analysis revealed no difference in session performance compared to session 1 (Fig. 5.D). Finally, there was a main effect of session on initiation time (F_(2.853, 45.65)_ = 5.448, p = 0.0032). Post-hoc analysis revealed young rats showed a decrease in initiation time at day 6 and day 7 compared to day 1 (p = 0.00253 and p = 0.0167 respectively). There was no effect of age or age*session interaction (p > 0.05) (Fig. 5.E).

A summary of behavioural results and their comparison with previously published behavioural data from aged mice is provided in Table 3.

## 4 Discussion

In this study we used a battery of behavioural tests hypothesised to map onto different behavioural dimensions of apathy syndrome, including motivational, emotional and cognitive components. Use of two operant effort-based decision making tasks found a trend towards an increase in physical effort-based responding in aged rats but no overall difference. However aged rats did show a reduction in spontaneous object exploration. Aged rats showed a greater latency to consume food in the novelty suppressed feeding task (NSFT) and preference for exploration of the side versus middle object in a position-based exploration test consistent with a higher level of anxiety-like behaviour rather than emotional blunting. Aged rats showed a corticosterone response to a restraint stressor equivalent to younger rats suggesting increased emotional reactivity is restricted to the behavioural level. Aged rats showed a profound impairment in reversal behaviour in a bowl digging version of the probabilistic reversal learning test. The impairment appeared specific to a loss in win-stay but not lose-stay behaviour and suggests perseverative behaviour. This impairment was not driven by changes to core affective state or reward-related cognition (reward learning assay (RLA)) or changes in reward sensitivity (sucrose preference test (SPT)). Of note, the operant version of this task did not pick up these same differences. Here, we consider how these behavioural findings relate to the psychiatric syndrome apathy and findings in aged mice [24] and consider the overall suitability of the use of aged rats as a model of apathy.

### 4.1 Aged rats show intact physical effort-based decision making

Patients with apathy exhibit a deficit in motivation, including some disruption to effort-based decision making [27]. The effort for reward (EfR) task and progressive ratio (PR) task are considered translational measures of effort based decision making [38,41,46,51]. These tasks have been shown to be sensitive to a range of manipulations that modulate motivational state in animals, including ageing in mice where a reduction in high effort, high value reward responding and an increase in low effort, low value reward engagement was found [24]. Deficits have also been found in patients with apathy associated with Huntington’s Disease [21] and neurocognitive disorders such as Alzheimer’s Disease and Parkinson’s Disease [12,56]. We found no evidence of impairment in aged rats, and in fact, there was a trend towards an increase in high effort choices in the aged rats, suggesting effort-based decision making is not affected by ageing in this species. A trend towards an increase in high effort responding may be linked to the potentially greater reward sensitivity observed in aged rats via the sucrose preference test, where a stronger preference for the sucrose solution compared to the younger rats was observed. The SPT is often used as a measure of anhedonia [43]. Apathy and anhedonia share overlapping characteristics relating to blunting in response to rewarding/pleasurable activities [22]. However, apathy is additionally characterised by a blunting in response to negative/aversive activities [25]. Here, SPT was used to assess the reward sensitivity component in combination with other tasks like the NSFT, position-based exploration test and restraint stress to also assess responsivity to aversive events. In this way, apathy may be distinguished from anhedonia.

These findings relating to intact or heightened effort-based decision making may in part be driven by behavioural context. It has previously been shown that, in contrast to other effort-based behaviours, instrumental lever press responding is conserved in aged rats [42]. Therefore, in addition to assessing motivated behaviour in a conditioned responding context, we assessed motivation to explore novel objects. Previous work has shown that exploration of novelty is a motivated behaviour based on an intrinsic drive to forage and seek reward. Novelty-based motivation has been found to be sensitive to ageing at in humans and rodents [14] and has also been shown to be reduced in patients with apathy (Yamagata et al., 2004, Kaufman et al., 2016). We found that aged rats exhibit a robust reduction in exploration of novel objects, suggestive of a motivational impairment in this context. Thus, in the context of age-related behavioural phenotyping, operant-based conditioning methods may be less sensitive to motivational deficits in this species and more ethologically relevant, forage-based methods may provide a more sensitive readout of motivational state in rats.

### 4.2 Aged rats show heightened affective reactivity inconsistent with emotional blunting

Patients with apathy often exhibit emotional blunting symptoms, where they show limited reactivity to environmental stimuli [28]. In animal models, this can be quantified by assessment of behavioural and physiological response to behaviours relating to the negative valence domain [24,25], where a reduction in appropriate response is indicative of affective blunting. The NSFT is a commonly-used measure of behaviours relating to anxiety, where the rodent must overcome its aversion of a novel, open space to eat from a bowl placed in the center [7]. We found that aged rats took longer to eat from a centrally placed bowl, which is indicative of heightened anxiety-like behaviour [7]. Age-related metabolic differences have the potential to confound appetitive tasks. We found that aged rats consumed more lab chow in a consumption test and therefore loss of appetite does not explain increased latency to consume food. However we cannot fully rule out age-related metabolic differences driving at least part of this behaviour. Therefore, to assess anxiety-like behaviour in a non-food motivated context, we developed a position-based exploration test based on the well-characterized principle that a rodent will exhibit thigmotaxis when placed in an open arena [20]. As such, the rodent will bias its exploration of an object positioned to the side of the arena versus the object placed in the center. A stronger exploration bias may indicate a greater level of anxiety-related behaviour. Aged rats showed a stronger bias for exploration of the object placed at the side rather than the center of the arena. Thus, consistent with the results from the NSFT, aged rats show increased anxiety-like behaviour. Aged rats exhibited a similar increased corticosterone response to restraint stress to that of younger rats, suggesting this increase in reactivity is conserved to the behavioural level. These findings contrast with our prediction of a blunted response and our findings in aged mice, where a blunted restraint-induced corticosterone response was observed and reduced anxiety-related behaviour in the NSFT, indicative of emotional blunting [24,34]. These findings further highlight key species differences in behaviours relevant to apathy with no evidence of emotional blunting observed in aged rats but an increase in anxiety-related behaviours.

### 4.3 Aged rats show a reduction in cognitive flexibility but only in a bowl-digging-based task

Impairments in cognitive flexibility have been found across conditions where apathy commonly occurs [33]. The rodent PRLT is usually conducted as a touchscreen assay [10,13,54], which is characterised by extended training periods, large numbers of trials and repeated sessions which may result in performance becoming more dependent on procedural learning strategies. Bowl digging assays may provide more ethologically relevant learning and have been used previously to assess affective state, attention and cognitive flexibility [6,31,47]. Here, we found most rats successfully learned to select the rewarded substrate and while there was no group difference, there was a trend towards aged rats taking more trials to learn in the acquisition phase. Strikingly, when the rule was reversed, all aged rats were unable to reverse, consistent with an impairment in cognitive flexibility. Assessment of likelihood that the rat would choose the previously rewarded bowl (win-stay probability) showed that while win-stay behaviour was equivalent to younger rats in the acquisition phase, it was markedly reduced in the reversal phase. This deficit was not seen in lose-shift behaviour, the likelihood the rat will choose the other substrate on the next trial after it was unrewarded on the previous trial. Therefore, this reversal learning impairment is likely due to a failure to remove prior associations with a positive cue, known as ‘perseverative’ behaviour [33]. Perseverative behaviour in old age has been widely reported in other rodent studies. Aged rats could learn normally but showed impaired reversal in a spatial T maze discrimination task, odour discrimination task and visual discrimination task, showing this behaviour is consistent over multiple sensory domains [9,45]. Elderly humans also show impairments in reversal learning and a mild impairment in acquisition learning compared to younger adults [53].

These changes were not driven by differences in core affective state or reward-related cognition, as revealed by the RLA, where both young and aged rats showed a robust and equivalent reward-induced positive bias. This task has been shown to be a sensitive measure of reward-related cognition and core affective state changes, where models such as early life adversity, chronic pain and chronic administration of pro-depressant drugs impairs reward learning [37,48,49]. These changes were also not driven by differences in reward sensitivity, as aged rats showed an intact and even enhanced preference for 2% sucrose solution over standard tap water in the SPT. Thus, this appears to be an impairment specific to cognitive flexibility.

Of note, we do not observe this same deficit in cognitive flexibility either in terms of absolute reversals or win-stay probability in the touchscreen version of the PRLT. Here, we observed similar levels of performance between groups across sessions. It has been suggested that ethological relevance of the cue or context can impact on within-session learning. For example, it has previously been shown that rats learned an odour discrimination reversal task more quickly than a visual version of the task [9]. While visual processing is a dominant sense in humans, this is not the case for rodents [26]. Therefore, the operant version of the PRLT may have reduced sensitivity to parse age-related differences in cognitive flexibility. Specifically, by using more ethologically relevant cues (digging in substrate vs visual/spatial cue) rats may form stronger associations between cue and reward which require greater attention to reward feedback to unlearn. Here, a deficit may more easily be observed.

### 4.4 Species differences in apathy-related behaviour: potential causes and implications for apathy research

The differences in age-related behavioural changes between species highlights the importance of considering these differences before choosing a rodent model for the study of apathy-related behaviour. Differences in behavioural changes between species has been highlighted in other work. A study found discrepancies between a HD mouse and rat model, which share the same genetic construct (bacterial artificial construct (BAC)-HD). While BAC-HD mice showed an increase in fear conditioning behaviour [2], BAC-HD rats showed a reduction in the same paradigm [1]. This further emphasises the importance of comparing models when attempting to ask a specific question relating to behavioural research. There are several potential explanations for this species difference in the behavioural effects of ageing, 1) differing basal levels of anxiety between species and 2) different relative ages between species. It has previously been reported that mice experience more stress and anxiety than rats in the laboratory setting [15]. Differences in task experience induced by fundamental differences in anxiety-related behaviour may drive conflicting age-related behavioural changes between species, across multiple domains of apathy. Differences in lifespan between the rat and mouse make it difficult to make direct comparisons between species [44,50]. While both species were tested in a window considered to be ‘aged’, the length of the aged phase may differ between species. This may induce variation in when age-related impairments develop. It is also not known the extent to which the laboratory environment and associated breeding has impacted on ageing in these species.

It should be noted that the tests in this work only focused on behaviours relevant to the behavioural-cognition and emotional domains of apathy and did not consider the social domain of apathy. It may be that considering the generally collaborative and complex nature of human social behaviour, and the greater levels of social interaction and hierarchy complexity in rats, that the rat may be more appropriate than mice to study the social domain of apathy. The inclusion of an additional domain of apathy to the behavioural battery could reveal further species differences, allowing a more informed choice of which species to use for the apathy domain of interest.

### 4.5 Other considerations

It is important to note that the use of aged females would improve overall translatability of findings, though they were not made available to us due to increased risk of age-related impairments. Due to limited data availability, it is unclear whether aged females would show a similar pattern of deficits in behaviours relating to apathy however there is some limited evidence for differences in motivated behaviour [52]. It should also be noted that while our sample sizes were selected to detect larger effects, we may have missed more subtle changes in age-related behaviour. A larger sample size could also allow for additional important questions to be asked relating to individual consistency in performance across tasks.

## 5 Conclusion

We found limited evidence for effort-based motivational deficit or emotional blunting in the rat but observed an increase in anxiety-related behaviours and impairments in cognitive flexibility in a bowl-digging reversal learning task. The findings in aged rats were very different from those observed in aged mice across these different behavioural domains suggesting there are key species difference in apathy-relevant behavioural changes associated with ageing. The evidence for a reduction in cognitive flexibility and perseverative behaviour in aged rats may be useful in relation to understanding more about age-related cognitive flexibility and the use of a more ethologically relevant task seems to increase sensitivity to these impairments when compared to a touchscreen task with prolonged training periods and repeated exposure to both acquisition and reversal learning phases. However, this does not address the challenge of studying apathy based on impairments across multiple domains. The behavioural impairments observed in mice suggested both motivational impairments and emotional blunting develop in ageing, but evidence of cognitive impairments in a novel object task was not observed. It should be noted that this task measures recognition memory and is not sensitive to changes in cognitive flexibility. In contrast, aged rats showed either no change or an increase in motivation in effort-related decision-making tasks which were not related to appetite. They also exhibited an increase in anxiety-related behaviours. Overall, these studies do not support the use of an aged rat model (21–27 months) to study the domains of apathy tested. However, aged mice may represent a more translationally relevant model and further evaluation of their cognitive flexibility would be useful. It might also be interesting to investigate further the species differences seen between rats and mice and expand the behavioural characterization of ageing in these two important species.

## Supplementary Material

**Appendix A. Supporting information**

Supplementary data associated with this article can be found in the online version at doi:10.1016/j.bbr.2024.114977.

Supplementary material 1

Supplementary material 2

## Figures and Tables

**Table 1:** Summary of behavioural tasks used, the associated Research Domain Criteria behavioural construct tested [11,25] and hypothesised mapping to apathy symptom domains according to the 2018 consensus group classification [39]. Apathy syndrome consists of behavioural changes relating to multiple behavioural domains including ‘emotion’, ‘behaviour-cognition’ and ‘social motivation’. In patients, behaviours relating to the emotion domain include a blunted or lack of reaction to an external piece of news (positively or negatively valanced). Behaviours relating to the behaviour-cognition domain include reduced motivation to start/finish a task. Behaviours relating to the social motivation domain include a reduction or lack of initiation of social contact [3].

Behavioural task (rodent)	Behavioural/physiological construct(s) measured (Research Domain Criteria)	Hypothesised mapping to apathy domain (2018 international consensus group classification)
Position-based object exploration	Spontaneous exploration, anxiety-like behaviour	Behaviour-cognition Emotion
Novelty supressed feeding test	Anxiety-like behaviour	Emotion
Effort for reward	Motivation for reward	Behaviour-cognition
Progressive ratio	Motivation for reward	Behaviour-cognition
Restraint stress	Hormonal reactivity to stress	Emotion
Reward learning assay	Reward learning, core affective state	Emotion
Sucrose preference test	Reward sensitivity	Emotion
Probabilistic reversal learning (bowl digging)	Cognitive flexibility, feedback sensitivity	Behaviour-cognition
Probabilistic reversal learning (touchscreen)	Cognitive flexibility, feedback sensitivity	Behaviour-cognition

**Table 2:** Key output measures for the probabilistic reversal learning task (bowl digging and touchscreen).

Output measure	Description	Calculation
Reversals	The number of times the rat learns the new rule following reversal of contingencies.	
Win-stay probability	The probability that the rat will choose the bowl where it found a reward in the previous trial.	Win-stay probability = win-stay count / (win-stay count + win-shift count)
Lose-shift probability	The probability that the rat will shift its choice to the other bowl after not finding a reward in the previous trial.	Lose-shift probability = lose-shift count / (lose-shift count + lose-stay count)

**Table 3:** Summary of behavioural changes in the aged animal versus the young animal. NC = no change. ND = not determined, test was not conducted in that species. Blue-behaviours relating to behaviour-cognition domain (motivation), peach- behaviours relating to emotional domain, yellow- behaviours relating to behaviour-cognition domain (cognition).

Behavioural task/physiological measure	Rat	Mouse (Jackson et al., 2021)
Position-based object exploration: total exploration	Reduced	ND
Position-based object exploration: position bias	Increased	ND
Novelty suppressed feeding test	Increased latency to feed	Decreased latency to feed
Effort for reward	NC	Decreased trials and chow consumed
Progressive ratio	NC	Decreased final ratio achieved
Hormonal response to restraint stress	NC	Decreased levels
Reward learning assay	NC	ND
Sucrose preference test	Increased preference	Decreased preference
Probabilistic reversal learning (bowl digging)	Decreased reversals	ND
Probabilistic reversal learning (touchscreen)	NC	ND

## Data Availability

Data will become available following publication via a link to the Open Science Framework https://osf.io/7gdfa/.

## Abbreviations

CORT: corticosterone
CS: conditioned stimulus
EfR: effort for reward
FR: fixed ratio
NSFT: novelty supressed feeding test
PR: progressive ratio
PRLT: probabilistic reversal learning task
RIA: radioimmunoassay
RLA: reward learning assay
RM: repeated measures
SPT: sucrose preference test
