# Supplementary material for: Characterisation of behaviours relevant to apathy syndrome in the aged male rat
Source: Behav Brain Res. Author manuscript; Available in PMC 2025 Jul 4. (PMC7617836; doi:10.1016/j.bbr.2024.114977)
Supplement: Supplementary material 1 [file EMS206159-supplement-Supplementary_material_1.docx]

**Supplementary figures**


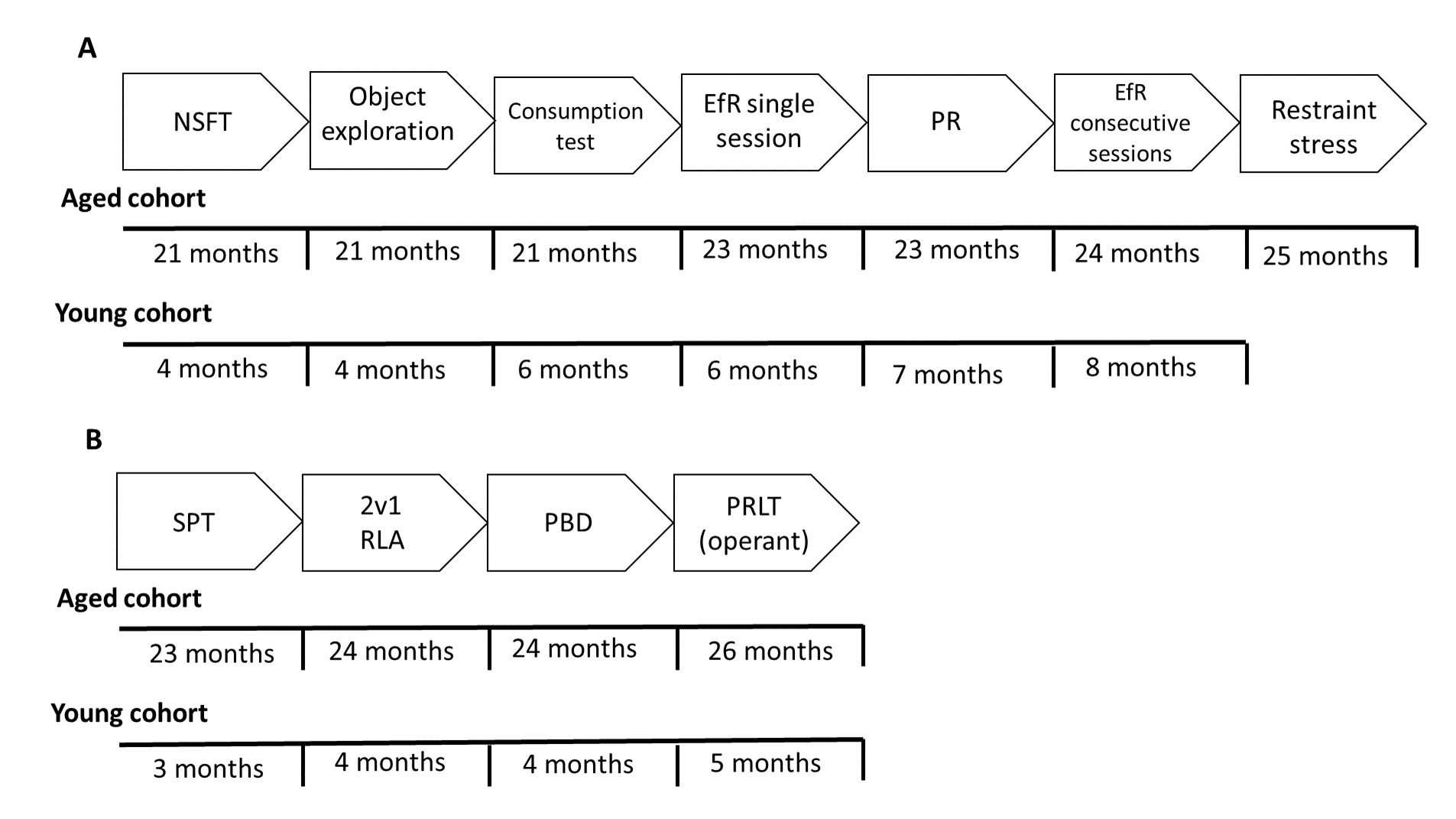
**S1**

***S1. Timeline for analysis of age-related behaviours.*** *Two cohorts of rats underwent a series of behavioural tasks.* ***A*** *Cohort 1.* ***B*** *Cohort 2. NSFT-novelty supressed feeding test, EfR- effort for reward, PR-progressive ratio, SPT- sucrose preference test, RLA-reward learning assay, PBD-probabilistic bowl digging, PRLT-probabilistic reversal learning.*

**S2**

| Experiment | Measure | Statistical exclusion in dataset |
| --- | --- | --- |
| Reward learning assay | Reward bias | N = 1 young, n = 1 aged outliers. |
| Probabilistic bowl digging | Trials to first rule | None |
|  | Number of reversals | None |
|  | Win-stay probability | None |
|  | Lose shift-probability | N = 1 young, acquisition phase |
|  | % Sucrose preference | N = 1 aged |
| Progressive ratio | Food res- final FR completed | N = 1 young |
|  | Food res-breakpoint | None |
|  | Ad lib- breakpoint | None |
| Effort for reward | First test-trials | N = 1 young |
|  | First test-chow | None |
|  | Food res-trials | N = 1 young, n = 1 old full exclusion for both |
|  | Food res-chow | N = 1 aged (session 5) |
|  | Ad lib-trials | N= 1 young (session 5), n = 1 aged full exclusion |
|  | Ad lib-chow | N = 1 aged (session 2) |
| Probabilistic reversal learning (operant) | Trial first rule was learned | N = 2 young (session 4, 7)  N = 1 aged (session 6) |
|  | Reversals | N = 1 young (session 8) |
|  | Win-stay | N = 3 young (Session 4, 5, 6), n = 1 aged (session 3). |
|  | Lose-shift | N = 4 young (session 1, 2, 3, 7) and n = 1 aged, full exclusion |
|  | Initiation time | N = 2 young (session 3 and full exclusion) and n = 2 aged (session 1 and full exclusion). |
| Novelty supressed feeding test | Latency to approach | None |
|  | Latency to eat | None |
|  | Amount consumed | None |
| Restraint stress | Plasma CORT | N = 1 young (missing value) |
| Position based object exploration | Time spent exploring | N = 1 aged |
|  | Bouts of exploration | N = 1 young, n = 1 aged |
|  | % preference for side | N = 1 young |

***S2.*** *Summary of excluded data points across experiments according to criteria outlined in methods.*

**S3**


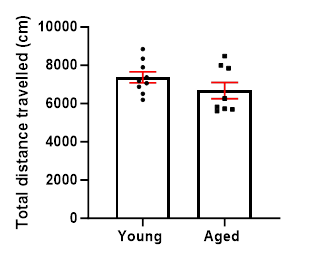


***S3.*** *There was no difference between age groups in total distance travelled in an open field arena (cm). Activity in the open field was recorded for 15 min under red lighting and locomotion was analysed using Ethovision.*


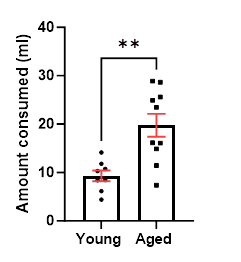
**S4**

***S4.*** *Aged rats consumed more liquid overall in the sucrose preference test. **P < 0.01.*
